# Associations between Multimorbidity Clusters and All-Cause Mortality, Quality of Life and Physical Function: A Systematic Review Protocol

**DOI:** 10.12688/hrbopenres.14205.2

**Published:** 2026-07-10

**Authors:** Joice Cunningham, Claire M. Buckley, Patricia M. Kearney, Ciara Kelly, Leonard Browne, Emma Connolly, Rehab Elhiny, Edward W. Gregg, Jennifer Martin, Sarah M. O’ Brien, Elizabeth Oluwaseyi Bodunde, Kate N. O’ Neill, Valeria Lima Passos, Diarmuid Quinlan, Prachi Sharma, Susan M. Smith, Austin G. Stack, Emma Wallace, Linda M. O’Keeffe

**Affiliations:** 1 School of Public Health, University College Cork, Cork, County Cork, Ireland; 2 Public Health, National Health Service Improvement Team, Health Service Executive, Dublin, Ireland; 3 University of Limerick School of Medicine, Limerick, County Limerick, Ireland; 4 Trinity College Dublin, The Irish Longitudinal Study on Ageing, Dublin, Ireland; 5 Discipline of Medical Gerontology, School of Medicine, Trinity College Dublin, Dublin, Leinster, Ireland; 6 School of Pharmacy, University College Cork, Cork, County Cork, Ireland; 7 School of Population Health, RCSI University of Medicine and Health Sciences, Dublin, Ireland; 8 Health Service Executive, National Quality & Patient Safety Directorate, Dublin, Ireland; 9 Health Service Executive, Office of the National Clinical Advisor and Group Lead for Chronic Disease, Clinical Design & Innovation, Office of the Chief Clinical Officer, Dublin, Ireland; 10 School of Pharmacy and Biomolecular Sciences, RCSI University of Medicine and Health Sciences, Dublin, Ireland; 11 15 Hogan Place, Irish College of General Practitioners, Dublin, Ireland; 12 Department of General Practice, University College Cork, Cork, Ireland; 13 Discipline of Public Health & Primary Care, School of Medicine, Trinity College, Dublin, Trinity College, Dublin, Ireland; 14 Department of Nephrology and Internal Medicine, University Hospital Limerick and University of Limerick School of Medicine, Limerick, Ireland; 15 MRC Integrative Epidemiology Unit at the University of Bristol, University of Bristol, Bristol, UK; 16 Population Health Sciences, Bristol Medical School, University of Bristol, Bristol, UK

**Keywords:** multimorbidity, multiple long-term conditions, clusters/patterns, all-cause mortality, quality of life, physical function

## Abstract

**Background:**

Multimorbidity, defined as the coexistence of two or more chronic health conditions in affected individuals, is recognised as a significant global public health concern. While previous research has identified common multimorbidity clusters, the relationships of these clusters with health outcomes is not well understood.

**Aim:**

To systematically retrieve, synthesise, and appraise the available evidence on the association between multimorbidity clusters and mortality, quality of life (QoL), and physical function.

**Methods:**

We will conduct a systematic search of PubMed, EMBASE, Web of Science, Ebsco APA PsyInfo and CINAHL from inception until April 2025. Eligible studies will include observational studies (e.g. cohort, cross-sectional or case control) investigating the association between multimorbidity clusters and all-cause mortality, QoL, or physical function in community- dwelling adult populations. Multimorbidity clustering will be defined as the non-random co-occurrence of chronic health conditions. Outcomes of interest include all-cause mortality, health-related quality of life (QoL) (i.e., self-perceived health status), and physical function (e.g., functional independence/physical performance)). Risk of bias will be evaluated using appropriate tools, e.g., the risk of bias in observational studies of exposures (ROBINS-E) tool. Findings will be synthesised narratively, and if feasible, a meta-analysis will be performed. Given the anticipated heterogeneity of multimorbidity clusters, a robust methodological framework, informed by existing multimorbidity literature and stakeholder engagement, will be applied to facilitate comparability across studies.

**Conclusion:**

This review will lay out and summarise current evidence on the association between different multimorbidity clusters and key health outcomes, including all-cause mortality, QoL, and physical function. It will address methodological approaches used to investigate the multimorbidity-health outcomes associations of interest and summarise current evidence. The findings will enhance our understanding of the unique burden imposed by different clusters of chronic conditions and may inform improvements in healthcare policy, the delivery of health services and patient care. Prospero Registration Number: CRD420251014288 (Date of Registration: 25 Jun 2025 13:08 UTC version 1.0 published).

## Background

Multimorbidity, or “multiple long-term conditions”, defined as the coexistence of two or more chronic conditions in the same individual, is a significant global public health challenge, placing substantial burden on individuals and on healthcare systems. ^1^ The World Health Organization (WHO) defines chronic conditions as “health problems that require continuous management over a period of years or decades”, highlighting the prolonged duration of these conditions and the necessity for ongoing care to manage symptoms, prevent complications, and enhance the quality of life (QoL) for individuals living with them. ^2^ It is estimated that 37% of adults worldwide are living with multimorbidity, with over 50% of individuals aged 60 years and over affected. ^3^ The high prevalence of multimorbidity in contemporary populations reflects advances in healthcare, population ageing, and adverse trends in risk factors for chronic disease, amongst other factors. ^4^ Multimorbidity is associated with a range of adverse health consequences, including greater disability, poorer QoL, polypharmacy, and higher mortality. ^4^ Individuals living with multimorbidity have complex healthcare needs, requiring increased healthcare utilisation and expenditure, which contributes to significant strain on healthcare systems worldwide. ^5^ Despite this, healthcare systems, clinical practice guidelines, and healthcare provider training have traditionally been designed to manage individual chronic conditions. This single-disease paradigm fails to address the multifaceted needs of individuals with multimorbidity and has proven inadequate in delivering effective patient-centred care. ^6^ As a result, individuals with multimorbidity often experience fragmented, uncoordinated, and sometimes conflicting care recommendations, exacerbating their treatment burden and leading to suboptimal health outcomes. ^7^

Historically, multimorbidity has been measured simply by counting the number of chronic conditions an individual has, with equal weight assigned to each condition in the “multimorbidity score”. ^8^ While this approach is straightforward, it fails to account for how specific combinations are associated with overall health outcomes. ^9^ Previous systematic reviews have shown that multimorbidity is associated with poorer outcomes, including higher mortality risk and reduced QoL. ^10^ ^,^ ^11^ However, these studies primarily examined the association of number of conditions with outcomes, rather than exploring how specific combinations of conditions are associated with outcomes. In contrast, the concept of multimorbidity clustering focusses on the non-random co-occurrence of conditions. ^12^ While there is variability in the multimorbidity clustering literature, some consistent clusters (e.g. cardiometabolic clusters of diabetes, hypertension, and cardiovascular diseases) have been replicated, believed to have shared underlying biological pathways and risk factors. ^13^ ^,^ ^14^ Understanding which conditions tend to cluster together and examining the associations of these clusters with health outcomes has been identified as a key research priority. ^15^ Moreover, the associations of multimorbidity clusters on health outcomes are not uniform across all individuals. Increasingly, it is recognised that demographic factors such as age, gender, and socioeconomic status may modify the relationship between multimorbidity clusters and health outcomes. ^16^ A better understanding of the unique burden imposed by specific multimorbidity clusters on health outcomes is likely to provide new insights for developing more targeted and patient-centered healthcare delivery models that more effectively address the needs of individuals. ^10^ Furthermore, mortality and HRQoL have been identified as essential core outcomes for multimorbidity research. ^17^

## Study aim

The aim of this review is to systematically retrieve, synthesise and appraise the available evidence quantifying the association between multimorbidity clusters and all-cause mortality, QoL, and physical function.

## Methods

The reporting of this systematic review protocol is based on the Preferred Reporting Items for Systematic Reviews and Meta-Analyses Protocols (PRISMA-P) 2015 checklist) ^18^ can be found in the online supplementary material (see *Extended data*). ^19^ Furthermore, this review is registered with the International Prospective Register of Systematic Reviews (PROSPERO) (registration number: CRD420251014288).

### Eligibility criteria

The PECOs (Population, Exposure, Comparison or Control, Outcomes and Study Design) framework was used to structure the eligibility criteria ^20^ [ Table 1].

**Table 1.** Inclusion and exclusion criteria based on modified PECoS.

PECoS	Inclusion Criteria	Exclusion criteria
*Population*	Studies that examine the general population in which groups or subgroups of participants are comprised of adults aged ≥18 years and older.	Studies including exclusively paediatric (<18 years of age) populations. Studies that include populations selected based on having a principal condition of focus (e.g., studies including only adults with diabetes). Studies that focus on nongeneral populations (e.g., individuals who are hospitalised or residing in long-term care facilities) or individuals with specific health contexts (e.g., pregnant people or homeless individuals).
*Exposure*	Studies that use data-driven methodology to classify conditions into multimorbidity clusters without any single condition focus.	Studies that that use non-data-driven approaches to assign conditions to clusters (e.g. studies that analyse the number of conditions without using statistical techniques to detect non-random disease associations). Studies that include fewer than ten conditions.
*Comparator*	Studies including single conditions or no conditions.	n/a
*Outcomes*	Outcomes including all-cause mortality, QoL (e.g., Short Form 36 Health Survey (SF-36), and physical function (e.g., Timed Up and Go test)).	Condition-specific measures (i.e., outcomes designed for specific diagnostic groups or patient populations).
*Study Type/ Design*	Original quantitative research (e.g. cohort, cross-sectional or case-control study designs). No restrictions on geographical location or language.	Interventional studies, qualitative research, literature reviews or meta-analyses. Non-peer reviewed articles (e.g. editorials, commentaries, case studies and opinion pieces). Conference abstracts without full-text availability or study protocols.

### Population

We will include studies that include general population cohorts in which groups or subgroups of participants are comprised of adults aged 18 years and older. Studies must involve community-dwelling adults, defined as adults who are not living in a long-term care institution. ^21^ If a relevant study is identified which includes individuals younger than 18, it will only be included if separate results are provided for those aged 18 and older. Included studies must investigate multimorbidity, defined as the co-occurrence of two or more chronic conditions within an individual, without focusing on any single index condition. We will exclude studies that focus solely on paediatric populations, individuals who are hospitalised, or those residing in long-term care institutions. Where studies are conducted in primary care settings, they will be eligible for inclusion provided they use a population-based data source, such as electronic health records drawn from a representative population, or a study explicitly using a representative sampling frame. Studies that select participants based on a prespecified index condition (e.g., multimorbidity among individuals with diabetes) will be excluded, as these studies focus on comorbidity rather than multimorbidity. Comorbidity refers to any distinct additional health condition that exists or may occur in a patient with a specific index condition. ^22^ While individuals with comorbidity also have multimorbidity, the sample in these studies is unlikely to be representative of the broader population with multimorbidity, as the index condition and its frequently co-occurring conditions will be overrepresented. ^23^ Additionally, studies restricted to specific health-related contexts, such as homeless individuals, veterans, or pregnant people, will also be excluded due to limited generalisability.

### Exposure

We will include studies that investigate multimorbidity, defined as the co-occurrence of multiple chronic conditions in an individual, without focusing on any single condition. There is considerable variability in how multimorbidity is defined and operationalised in the literature. ^15^ Multimorbidity is typically defined as the coexistence of two or more chronic conditions. ^24^ While there is a lack of a single, universally accepted definition for chronic disease, ^25^ common characteristics include the non-self-limiting nature of conditions, an association with persistent and recurring health problems, and a duration, measured in months and years rather than days and weeks. ^26^ Studies must employ data-driven methodology to identify and classify conditions into multimorbidity clusters or combinations. Examples of such methods include latent class analysis, hierarchical clustering, and network analysis. The statistical methods used to identify the clusters must be clearly described. Studies that apply non-data-driven approaches or analyses based solely on condition counts, without using statistical techniques to detect non-random disease associations, will be excluded. The distinction between comorbidity and multimorbidity is important, particularly concerning patient sampling. ^23^ Studies must include a minimum of ten distinct conditions in their clustering analyses, to be included. A minimum disease list of this size is required to produce stable and interpretable clusters: analyses using fewer conditions risk deriving artefactual groupings driven by chance co-occurrence rather than underlying biological or clinical pathways. This threshold is based on previous recommended cut-offs, where studies typically include between ten and twelve conditions to enhance the interpretability and stability of the clusters. ^13^ ^,^ ^27^ Studies that include fewer than ten conditions in their clustering analyses will be excluded.

### Outcomes

The outcomes of interest for this review include all-cause mortality, QoL (including health-related quality of life (HRQoL)), and physical function. QoL is multi-dimensional and relates to an individual’s general well-being status in relation to their value, environment, cultural and social context in which they live. ^28^ While HRQoL is the self-perceived health status of individuals and includes physical, mental, emotional, and social domains. ^29^ Measurement of physical functioning may range from self-report questionnaires (e.g., The Barthel Index, which measures functional disability in activities of daily living), to performance measures (e.g., The Berg Balance Scale, which assesses static balance and fall risk). Generic scales are preferred when measuring health outcomes in people with multiple conditions or comorbidities. ^30^ Therefore, condition-specific measures (i.e., outcomes designed for specific diagnostic groups or patient populations) will not be eligible for inclusion. To be eligible for inclusion, studies must report outcomes specifically according to multimorbidity clusters.

### Study design/type

Original quantitative research reporting multimorbidity clusters and associated outcomes of interest will be eligible for inclusion (e.g., cohort, cross-sectional, and case-control study designs). Other study designs containing quantitative data e.g. mixed method research will be considered if the quantitative results can be extracted. Interventional studies (e.g. randomized control trials), qualitative research, literature reviews or meta-analyses, non-peer reviewed articles (e.g., editorials, commentaries, case studies, and opinion pieces), and studies available only in abstract form will be excluded. In cases, where abstracts or protocols are retrieved, attempts will be made to access full texts by contacting the authors. There will be no restrictions on articles by publication date or language of publication. Where articles not in English are retrieved, translation will be done using online translation software.

## Information sources

The following databases will be searched for primary research studies: Scopus (PubMed/Medline), Embase, Web of Science, Ebsco APA PsyInfo, and CINAHL. Additionally, grey literature searching will include screening the reference lists of relevant systematic reviews and/or meta-analyses previously published.

### Search strategy

A comprehensive search strategy aimed at identifying relevant studies will be developed in conjunction with a medical librarian. Search terms will be based on the most relevant keywords, categories, and subheadings related to the research questions, including (1) multimorbidity, (2) clusters, (3) outcomes (all-cause mortality, QoL, HRQoL, and physical function), and (4) relevant study designs. Key words and index terms will also be identified from the title and abstract of relevant articles and will be used to inform the final search strategy. All search terms and keywords will be combined with the relevant medical subject headings (MeSH) for each of the databases using Boolean operators as appropriate. Following completion of the search, the reference lists of included articles will be used to identify additional relevant studies. An example of the search strategy for Ovid MEDLINE is shown in appendix 1.

### Study selection

Following the completion of the searches, the citations of identified studies will be imported to EndNote X9, and duplicates will be removed. Microsoft Excel and PICO Portal Software for Systematic Reviews will be used to perform screening. Two independent review authors (JC and RE or EOB) will independently screen titles and abstracts for eligibility according to the eligibility criteria outlined in Table 1. Following this, potentially eligible full texts will be independently evaluated by two review authors (JC and RE or EOB). Any disagreements will be resolved through discussion, and if consensus cannot be reached, a third review author (LOK) will act as an arbitrator.

## Data analysis and synthesis

### Data extraction

A pre-defined data-extraction form will be used to extract data from included studies. Data extraction will be then performed by JC and reviewed by LOK. Conflicts will be resolved by consensus between two or more researchers if required. Extracted data will include study characteristics (e.g., author(s), year of publication, study design, sample size and study setting), population characteristics (e.g., age, sex, and other sociodemographic details), characteristics of multimorbidity clusters (e.g., specific conditions used to identify disease clusters, measures of disease severity, and statistical methodology used for identification of clusters), outcomes (e.g., all-cause mortality, QoL, physical function), any relevant confounding factors considered, and risk of bias (methodological quality and risk of bias as determined by the relevant assessment tools).

### Quality appraisal

Articles included in this review will be assessed for methodological quality independently by two reviewer authors (JC and LOK) using appropriate tools based on study design (e.g., risk of bias in observational studies of exposures (ROBINS-E) tool and the Joanna Briggs Institute (JBI) checklist for cross-sectional studies).

### Certainty of the evidence (GRADE)

A ‘Summary of findings’ table will be used to draw conclusions about the certainty of the evidence. We will apply GRADE to assess the quality of the overall evidence for all outcomes. ^31^

### Synthesis

Review findings will be collated and summarised according to the review questions. The results of the literature search and study screening process will be presented in a PRISMA flow diagram, and extracted data will be presented in tabular form. Findings will initially be synthesised narratively. This will include structured summaries of study characteristics, multimorbidity cluster types, and direction/magnitude of associations with outcomes e.g. mortality. Findings will be grouped by study design (e.g., cross-sectional vs cohort), population type, and analytic method used for cluster identification. Significant heterogeneity is anticipated in terms of the multimorbidity clusters. We will consider methodological approaches used in previous reviews (e.g., grouping conditions by biological systems e.g., cardiovascular, metabolic, musculoskeletal, or using an a priori set of conditions) to ensure comparability across studies. If sufficient homogeneity exists across studies in terms of population, multimorbidity clusters, a meta-analysis will be conducted to estimate pooled effect sizes (e.g., hazard ratios, risk ratios, or odds ratios for mortality outcomes associated with multimorbidity clusters). For cohort studies, we will extract adjusted risk estimates with 95% confidence intervals. If studies are sufficiently similar in design and outcome measurement, we will use a random-effects model and assess heterogeneity using the I ^2^ statistic and Cochran’s Q test. Where appropriate, publication bias will be assessed using funnel plots and Egger’s test. Key stakeholder groups, including a patient advisory panel, will be involved to ensure the relevance of the findings. It is important to note that even clusters sharing the same label or similar composition may represent different underlying subpopulations, depending on the statistical method employed, the condition list used as input, and the characteristics of the study population. For studies using latent class analysis in particular, membership criteria and characteristic disease thresholds vary across studies, such that superficially similar clusters may reflect distinct clinical and demographic profiles. As specified in the Data Extraction section above, the statistical methodology used for cluster identification and the specific conditions included in clustering analyses will be extracted for each study. These variables will be considered when assessing the comparability of clusters across studies prior to any pooling decision. Where clusters are not deemed sufficiently comparable, meta-analysis will not be performed.

## Discussion

Multimorbidity represents a global health challenge. Existing literature has established that the interplay between chronic conditions is not merely additive but often synergistic and may be associated with poorer health outcomes. ^32^ While previous studies have shown that multimorbidity is linked to negative health consequences, such as increased disability, diminished HRQoL, and higher mortality risk, the interaction effect between different co-occurring conditions remain underexplored. Moreover, a condition count fails to explore whether specific combinations of conditions are responsible for driving poorer health outcomes. This systematic review aims to provide a more nuanced understanding of how specific multimorbidity clusters are associated with patient outcomes. By delineating which clusters pose the greatest threats to health, this review may inform the development of integrated care pathways that address the specific needs of patients with complex health profiles.

### Anticipated limitations

Several limitations are anticipated. First, substantial methodological heterogeneity is expected across included studies, arising from differences in clustering techniques (e.g., latent class analysis, hierarchical clustering, network analysis), the number and selection of conditions included in clustering analyses, and the stability of derived clusters. Second, considerable variation in the instruments used to measure QoL and physical function across studies may limit direct comparability of findings. Third, the predominant reliance on observational study designs means that residual confounding is an inherent limitation; unmeasured confounders may influence observed associations between multimorbidity clusters and health outcomes. Fourth, the inclusion of cross-sectional studies is an inherent limitation of this review and preclude causal inference.

## Ethics and consent

As the study protocol is for a systematic review of existing literature, ethical approval is not required.

## Ethics and dissemination

Upon completion of the review, the findings will be disseminated through multiple channels to ensure broad reach and impact:

Academic dissemination: The research findings will be presented at international scientific conferences to engage the academic community. Additionally, the results will be submitted for publication in a high-impact peer-reviewed journal relevant to the field, ensuring that the findings contribute to advancing knowledge in multimorbidity research.

Patient, Public, and Policy-oriented dissemination: To enhance the relevance and accessibility of the findings for patient, public, and policymaker audiences, a summary of key results will be developed in clear, non-technical language and disseminated to key stakeholder groups.

## Data Availability

Open Science Framework: This project contains the following extended data: Associations between Multimorbidity Clusters and All-Cause Mortality, Quality of Life and Physical Function: A Systematic Review Protocol: https://doi.org/10.17605/OSF.IO/6UESJ ^19^
− PubMed Search Strategy − PRISMA-P-checklist
