# Supplementary material for: Associations between Multimorbidity Clusters and All-Cause Mortality, Quality of Life and Physical Function: A Systematic Review Protocol
Source: HRB Open Res. 2026 Jul 10;8:100. Originally published 2025 Sep 10. [Version 2] doi: 10.12688/hrbopenres.14205.2 (PMC13673073; doi:10.12688/hrbopenres.14205.2)
Supplement: Peer review history

# Reviewer report: Reviewer response for version 2

**DOI:** 10.21956/hrbopenres.15988.r58196

**Published:** 2026-10-15

**Authors:** Ibrahim Alliu

**Affiliations:** 1 Georgia Southern University, Statesboro, Georgia, USA

The question is important, the protocol is clearly written, and the authors have responded constructively to the first round. My reservations are statistical and concern reporting. The most consequential issue is that the response to reviewers discloses that the review is largely complete, using analytic decisions that do not appear in the protocol. Several elements that determine whether any pooled or narrative estimate can be interpreted are also not yet specified.

 Points that must be addressed for the protocol to be scientifically sound: Major comments 1 to 6. Comments 7 to 9 and the minor comments are recommended.

 MAJOR COMMENTS

 1. Transparency about timing (Essential). The search was run in April 2025 and PROSPERO registration followed on 25 June 2025. The author response of 3 July 2026 states three things: the primary care criterion was revised during the conduct of the review, a harvest plot synthesis was performed instead of meta-analysis, and patterns were assigned to five overarching domains. Changing the eligibility criteria and synthesis plan after seeing studies is sometimes unavoidable, but a protocol's value lies in making this visible. Please do three things. (a) State in the Methods what stage the review had reached at registration and at each revision. (b) List every deviation from PROSPERO version 1.0 with its date and reason, and confirm that the PROSPERO record has been amended. (c) Describe in the protocol the harvest plot approach and the five domains actually used.

 2. Person-centred versus disease-centred methods (Essential). The eligible methods produce different exposures. Latent class analysis and k-means/k-medoids assign each individual to one class, so effects of class membership can be estimated directly. Factor analysis, hierarchical clustering of diseases, network analysis and observed-to-expected approaches group conditions rather than people. An individual can belong to several of these patterns or to none, and the association with an outcome is estimated through factor scores, pattern counts or thresholds set by the study authors. These estimates are on different scales. Please classify the eligible methods into these two families, state how the exposure is defined in each, and synthesise the two families separately, including on the harvest plots. Please also clarify whether frequency-ranked condition pairs or triads ("combinations") without a test of non-random co-occurrence are eligible.

 3. Comparator and reference category (Essential). Table 1 gives the comparator as "studies including single conditions or no conditions". This describes studies, not a comparison group. In practice, studies use different reference groups: people with no conditions, people without multimorbidity, a data-derived "healthy" or "non-specific" class, or everyone outside the index cluster. Effect sizes depend heavily on this choice and cannot be compared across different references. Please specify which reference definitions are acceptable, extract the reference group from each study, and state whether estimates with different references will be pooled, stratified or kept separate.

 4. Cluster composition versus disease burden (Essential). The review rests on the premise that specific combinations of conditions matter beyond the number of conditions. However, clusters usually differ in how many conditions their members have, and the "complex" class almost always has the worst outcomes. Unless studies account for condition count, an association between a cluster and an outcome is largely an effect of overall burden. For example, in a Cox model, log h_i(t) = log h_0(t) + Σ_k β_k C_ik + γ N_i + δ′Z_i, where C_ik indicates membership of cluster k, N_i is the number of conditions and Z_i are confounders. The coefficients β_k mean different things with and without the γ N_i term. Please extract whether each study adjusted for, stratified by or matched on condition count, and analyse count-adjusted and unadjusted estimates separately. Please also soften the Discussion's language on "synergy" and "interaction", because estimates of cluster membership do not identify statistical interaction between conditions.

 5. Statistical plan for quantitative synthesis (Essential). Please specify the following.

 (a) Effect measures. Hazard ratios, risk ratios and odds ratios should not be combined. For QoL, state whether you will use mean differences within a single instrument or standardised mean differences, and how the direction of effect will be aligned across instruments.

 (b) The estimator. Use REML or Paule-Mandel rather than DerSimonian-Laird, apply the Hartung-Knapp-Sidik-Jonkman adjustment given the small number of studies expected per cluster, and report 95% prediction intervals.

 (c) Dependence between estimates. Most studies report several clusters against one shared reference group, so their estimates are correlated. Use one estimate per study, a three-level model (theta_jk = mu + u_j + w_jk + e_jk) or robust variance estimation.

 (d) Overlapping cohorts. Data sources such as UK Biobank, SNAC-K, CPRD, SIDIAP and ELSA each yield several cluster papers. State how overlapping samples will be identified and which report will be retained.

 (e) Small-study bias. Assess funnel plots and Egger's test only where a comparison includes at least ten studies.

 (f) Effect modification. The Background states that age, sex and socioeconomic status may modify the associations, but no subgroup analysis or meta-regression is planned. Please prespecify these analyses or remove that rationale.

 6. Rules for cluster comparability and domain assignment (Essential). The whole synthesis depends on judging which clusters are comparable, yet no criteria are given. Please specify:

 (a) the five domains and the conditions that define each;

 (b) the rule for labelling a cluster, for example a within-cluster prevalence threshold, or an observed-to-expected ratio of at least 2 combined with exclusivity;

 (c) how mixed or non-specific clusters are handled;

 (d) whether assignment was done independently by two reviewers, with an agreement statistic.

 Without these, the harvest plots cannot be reproduced.

 7. Justification of the ten-condition minimum (Recommended). The new rationale says that fewer conditions risk groupings "driven by chance co-occurrence". Chance groupings are governed mainly by sample size, condition prevalence and how the clusters were validated (fit indices, entropy, bootstrap stability, replication), not by the length of the condition list. A short list mainly limits how many classes can be identified. Reference 27 (Fortin et al., 2012) recommends at least 12 conditions for estimating prevalence, not for clustering. Please revise the justification, cite sources that address clustering, and treat list length (10 to 212 in the included studies) as a prespecified variable in the synthesis.

 8. Risk of bias for cluster derivation (Recommended). ROBINS-E and JBI do not assess the quality of the clustering itself. Please add items on how the number of clusters was chosen, cluster stability and validation, handling of rare conditions, and whether clusters were derived in the same data used to estimate outcome associations. Please also prespecify a minimum confounder set (at least age, sex and a socioeconomic measure) and state how adjustment for possible mediators, such as frailty or disability, will be judged.

 9. Study design by outcome (Recommended). Cross-sectional studies cannot report mortality. Please state which designs are eligible for each outcome. Please also make explicit that reverse causation in cross-sectional studies of QoL and physical function will lead to downgrading in GRADE. The response to reviewers says this, but the revised text does not.

 MINOR COMMENTS

 1. Databases are inconsistent. The abstract lists PubMed, the Methods list "Scopus (PubMed/Medline)", the text cites an Ovid MEDLINE example in "appendix 1", and OSF holds a PubMed strategy. Scopus and PubMed are distinct databases.

 2. Please state when the update search will be run and that its date will be reported.

 3. Outcomes need clarifying. Does a single self-rated health item qualify as HRQoL? The Barthel Index is usually clinician- or proxy-rated, not a self-report measure. Do ADL/IADL disability, grip strength and gait speed count as physical function? How will multiple measures of the same outcome within one study be prioritised?

 4. Please list the extraction fields needed for the comments above: method family, number of clusters and how it was chosen, reference group, adjustment for condition count, covariates, follow-up and effect measure.

 5. Please update the GRADE reference (31). Cite Murad et al. (2017) for rating certainty without meta-analysis, the SWiM guideline (Campbell et al., 2020) for the narrative synthesis, and Ogilvie et al. (2008) for harvest plots.

 6. Please report in the PRISMA flow diagram how many studies were excluded for restricted populations (veterans, homeless people, pregnant people).

 7. Please describe the patient advisory panel's composition and the stages at which it contributes.

 8. The funding statement says no grants were involved, yet several authors are credited with Funding Acquisition. Please reconcile these.

Is the study design appropriate for the research question?

Partly

Is the rationale for, and objectives of, the study clearly described?

Yes

Are sufficient details of the methods provided to allow replication by others?

Partly

Are the datasets clearly presented in a useable and accessible format?

Yes

Reviewer Expertise:

Biostatistics; survival analysis; multimorbidity clusters and incident multimorbidity (MEPS); longitudinal and complex survey data analysis

I confirm that I have read this submission and believe that I have an appropriate level of expertise to confirm that it is of an acceptable scientific standard, however I have significant reservations, as outlined above.

# Reviewer report: Reviewer response for version 2

**DOI:** 10.21956/hrbopenres.15988.r56502

**Published:** 2026-07-21

**Authors:** Qi Feng

**Affiliations:** 1 The George Institute for Global Health, London, UK

I have no further comments.

Is the study design appropriate for the research question?

Yes

Is the rationale for, and objectives of, the study clearly described?

Yes

Are sufficient details of the methods provided to allow replication by others?

Yes

Are the datasets clearly presented in a useable and accessible format?

Yes

Reviewer Expertise:

multimorbidity, liver steatotic disease

I confirm that I have read this submission and believe that I have an appropriate level of expertise to confirm that it is of an acceptable scientific standard.

# Reviewer report: Reviewer response for version 1

**DOI:** 10.21956/hrbopenres.15623.r53531

**Published:** 2026-03-14

**Authors:** Carl Lavie, Charles Faselis

**Affiliations:** 1 John Ochsner Heart and Vascular Institute, New Orleans, USA; 2 Veterans Affairs Medical Center, USA and Stanford University School of Medicine, Palo Alto, CA, USA

Summary

 This protocol outlines a systematic review examining the association between data-driven multimorbidity clusters and key outcomes: all-cause mortality, quality of life (QoL) and health - related QoL, and physical function. The topic is important and timely and potentially very clinically relevant. This review focuses on identifying common patterns of diseases that occur together, rather than just counting diseases, and thus addresses a recognized gap in multimorbidity research(  refer 1; Lancet Multimorbidity Commission, 2018). The protocol is generally well structured and methodologically sound, with appropriate plans for risk-of-bias assessment (ROBINS-E, JBI), narrative synthesis, and potential meta-analysis.

 Major Comments

 1. Exclusion of primary care populations: The exclusion of studies conducted in primary care settings requires clarification. Much multimorbidity research is primary-care based (refer 2,3). Excluding these studies may substantially limit the evidence base and reduce generalizability.

 2. Exclusion of specific populations: Excluding veterans, homeless individuals, or other defined populations due to “limited generalizability” may introduce selection bias at the review level. These studies could instead be included and interpreted cautiously.

 3. Minimum threshold of ten conditions: Requiring ≥10 conditions for clustering analyses may exclude otherwise robust studies. The justification for this threshold should be clarified.

 4. Causal inference: Inclusion of cross-sectional studies limits interpretation, particularly for QoL and physical function outcomes. The discussion should clearly acknowledge reverse causation and residual confounding as important limitations.

 Strengths

 1. Clear distinction between multimorbidity and comorbidity (refer 1).

 2. Focus on data-driven clustering methods enhances conceptual consistency.

 3. Inclusion of core multimorbidity outcomes (mortality and HRQoL) aligns with consensus recommendations (Smith et al., 2018).

 4. Planned use of GRADE strengthens transparency.

 Anticipated Limitations

 The review is likely to face questions about:

 • Methodological heterogeneity.

 • Variation in clustering techniques and stability of clusters.

 • Differences in QoL and physical function instruments. • Residual confounding in observational studies.

 • Potential publication bias.

 These should be explicitly acknowledged.

 Overall Assessment

 This is a well-justified and potentially valuable systematic review protocol addressing an important gap in multimorbidity research that potentially provides clinically relevant information to influence clinical practice. Clarification of exclusion criteria and further detail on synthesis plans would strengthen methodological transparency. The review has strong potential to inform clinical practice and health policy , therefore , we await the results.

Is the study design appropriate for the research question?

Partly

Is the rationale for, and objectives of, the study clearly described?

Yes

Are sufficient details of the methods provided to allow replication by others?

Partly

Are the datasets clearly presented in a useable and accessible format?

Yes

Reviewer Expertise:

NA

We confirm that we have read this submission and believe that we have an appropriate level of expertise to confirm that it is of an acceptable scientific standard, however we have significant reservations, as outlined above.

## References

[1] Valderas JM et al. Ann Fam Med. 2009.

[2] Barnett K et al. Lancet. 2012. 10.1016/s0140-6736(12)60586-822509528

[3] Prados-Torres A et al. J Clin Epidemiol. 2014.

[4] Smith SM et al. Trials. 2018.

# Response

**Published:** 2026-07-03

**Authors:** Joice Cunningham

**Affiliations:** School of Public Health, University College Cork, Cork, County Cork, Ireland

**Author response to Major Comment 1: Exclusion of primary care populations**

 We thank the reviewer for raising this important point. On reflection, we agree that a blanket exclusion of primary care studies is difficult to justify, particularly given the centrality of primary care to multimorbidity research. We have therefore revised the protocol to remove this exclusion. However, during the conduct of the review, we encountered considerable variability in the scope and representativeness of primary care studies, and on reflection arrived at the following compromise: studies must use a population-based data source, such as electronic health records drawn from a representative population or a study explicitly using a representative sampling frame, regardless of the setting in which care was delivered. This will be assessed at the screening stage, prior to data extraction. Care setting will be recorded as a study characteristic and considered as a potential source of heterogeneity in the narrative synthesis.

**Author response to major Comment 2: Exclusion of specific populations (veterans, homeless individuals, pregnant people)**

 We thank the reviewer for this observation. We acknowledge that excluding these populations on grounds of limited generalisability may itself introduce selection bias at the review level, and we accept this as a limitation of the current review. We have therefore retained the exclusion criteria as originally specified in the protocol, and will note this as a limitation in the completed review. We will also flag this as a recommendation for future updates, wherein studies restricted to specific populations such as veterans, homeless individuals, or pregnant people could be included and their findings interpreted in the context of the specific population.

**Author response to major Comment 3: Minimum threshold of ten conditions**

 We thank the reviewer for prompting us to make this justification more explicit. We have maintained the minimum threshold of ten conditions in the protocol, but have expanded the relevant section to provide a clearer rationale. A minimum disease list of this size is required to produce stable and interpretable clusters: analyses using fewer conditions risk deriving artefactual groupings driven by chance co-occurrence rather than underlying biological or clinical pathways. The threshold is consistent with those applied in established systematic reviews in this field, which have typically ranged from ten to twelve conditions to ensure sufficient complexity for meaningful cluster detection. We acknowledge that this threshold inevitably excludes some studies, but consider it necessary to ensure methodological consistency across included studies.

**Author response to major Comment** **4: Causal inference and cross-sectional studies**

 We thank the reviewer for this important point. We have added a paragraph to the Discussion section of the revised protocol explicitly acknowledging this limitation. The revised text states that the inclusion of cross-sectional studies is an inherent limitation of the review, that cross-sectional designs preclude causal inference, and that findings from such studies should be interpreted with caution given the potential for reverse causation and residual confounding. The text further notes that these limitations will be acknowledged in the synthesis and will be considered when grading the certainty of evidence using GRADE.

# Reviewer report: Reviewer response for version 1

**DOI:** 10.21956/hrbopenres.15623.r53540

**Published:** 2026-02-28

**Authors:** Qi Feng

**Affiliations:** 1 The George Institute for Global Health, London, UK

This protocol for systematic review is well written with clear logic and sufficient details. The research question is well defined and urgently needed in multimorbidity research. My major concern is the heterogeneity in clusters identified in different studies. The potential sources of heterogeneity could include population, statistical method, and disease list.

 For latent class analysis, most studies used it to derive clusters of patients, and then to identify the characteristic diseases in each derived cluster, using various criteria. Therefore, even for seemingly the same “disease cluster” identified in multiple studies, they may actual represent different sub-population. I wonder whether the authors have any thoughts on this.

 I have one minor comment.

 (1) Abstract Methods: in search strategy, please extend the time limit to present, instead of April 2025 (I guess this was the time when the protocol was written).

Is the study design appropriate for the research question?

Yes

Is the rationale for, and objectives of, the study clearly described?

Yes

Are sufficient details of the methods provided to allow replication by others?

Yes

Are the datasets clearly presented in a useable and accessible format?

Yes

Reviewer Expertise:

multimorbidity, liver steatotic disease

I confirm that I have read this submission and believe that I have an appropriate level of expertise to confirm that it is of an acceptable scientific standard, however I have significant reservations, as outlined above.

# Response

**Published:** 2026-07-03

**Authors:** Joice Cunningham

**Affiliations:** School of Public Health, University College Cork, Cork, County Cork, Ireland

Author response to major concern (i.e., heterogeneity in clusters identified in different studies).

 We thank the reviewer for this highly pertinent observation. On reflection, we agree that this is a fundamental methodological challenge in this field and one that requires explicit acknowledgement and a clear analytic plan within the protocol. 

 We have added a paragraph to the Synthesis section of the revised protocol explicitly addressing this issue. The revised text clarifies that even clusters sharing the same label or similar composition may represent different underlying subpopulations, depending on the statistical method employed, the disease list used as input, and the characteristics of the study population. For studies using latent class analysis in particular, membership criteria and characteristic disease thresholds vary across studies, such that superficially similar clusters may reflect distinct clinical and demographic profiles. As already specified in the Data Extraction section of the protocol, the statistical methodology used for cluster identification and the specific conditions included in clustering analyses will be extracted for each study. The revised Synthesis section now makes explicit that these variables will be used when considering the comparability of clusters across studies prior to any pooling decision. Where clusters are not deemed sufficiently comparable, findings will be synthesised narratively, stratified by method and population type.

 As a side note, this issue was indeed encountered as we progressed with the review. In practice, substantial methodological heterogeneity was identified across included studies in pattern derivation methods, condition selection (ranging from 10 to 212 conditions), and pattern nomenclature. A narrative synthesis using harvest plots was ultimately performed rather than meta-analysis, with patterns assigned to five overarching domains to facilitate cross-study comparison. 

 Author response to minor comment: Search date in abstract

 We thank the reviewer for this comment. April 2025 was indeed the date on which the search was originally run. The search date in the abstract accurately reflects the date of the search as conducted, which is standard practice for systematic review protocols. Prior to the final review being submitted, the search will be rerun accordingly to bring it up to date. We have therefore retained April 2025 in the abstract at this stage, as this is the correct and accurate date of the search as conducted prior to the protocol submission.
